# Corneal Topographic and Aberrometric Measurements Obtained with a Multidiagnostic Device in Healthy Eyes: Intrasession Repeatability

**DOI:** 10.1155/2017/2149145

**Published:** 2017-01-04

**Authors:** David P. Piñero, Alberto López-Navarro, Inmaculada Cabezos, Dolores de Fez, María T. Caballero, Vicent J. Camps

**Affiliations:** ^1^Group of Optics and Visual Perception, Department of Optics, Pharmacology and Anatomy, University of Alicante, Alicante, Spain; ^2^Optometry Clinic, Prevention Service of the University of Alicante, Alicante, Spain

## Abstract

*Purpose*. To evaluate the intrasession repeatability of corneal curvature, eccentricity, and aberrometric measurements obtained with a multidiagnostic device in healthy eyes.* Methods*. This study enrolled 107 eyes of 107 patients ranging in age from 23 to 65 years. All of them underwent a complete anterior segment examination with the VX120 system (Visionix-Luneau Technologies, Chartres, France). Three consecutive measurements were obtained. The within-subject standard deviation (*S*_*w*_), intrasubject precision (1.96 × *S*_*w*_), and intraclass correlation coefficient (ICC) were calculated.* Results*. All *S*_*w*_ for corneal power measurements were below 0.26 D, with ICC above 0.982. The *S*_*w*_ for corneal astigmatism at different areas (3, 5, and 7 mm) was below 0.21 D, with ICC above 0.913. Concerning the axis of astigmatism, its *S*_*w*_ was below 11.27°, with ICC above 0.975. The *S*_*w*_ and ICC for corneal eccentricity were 0.067 and 0.957, respectively. The *S*_*w*_ and ICC for high-order aberration root mean square (RMS) were 0.048 *µ*m and 0.901, respectively. For 3rd- and 4th-order aberrometric parameters, all *S*_*w*_ were below 0.037 *µ*m and all ICC were higher than 0.84, except for quadrafoil RMS (ICC: 0.689).* Conclusions*. The multidiagnostic device evaluated is able to provide consistent measurements of corneal power, eccentricity, and third- and fourth-order aberrations in healthy eyes.

## 1. Introduction

Technological advances in ophthalmology have led to the development of multidiagnostic platforms that integrate several technologies in the same device allowing the measurement of different anatomical and optical parameters of the eye [1]. Specifically, these advanced devices provide an analysis of the shape and optical aberrations of the two surfaces of the cornea, a characterization of the distribution of corneal thickness, and even a volumetric analysis [1]. Concerning the geometric analysis of the anterior corneal surface, the combination of Scheimpflug imaging and Placido disk technologies has been demonstrated to be useful for obtaining consistent curvature and elevation data in normal healthy and even in keratoconus eyes [2–6]. Recently, a new multidiagnostic platform has been developed that provides automatic measurements of corneal topography, corneal, internal and ocular aberrations, pachymetry, anterior chamber depth, iridocorneal angle, pupil diameter under different luminance conditions, and intraocular pressure (IOP), which is the VX120 system (Visionix-Luneau Technologies, Chartres, France). This system combines Scheimpflug imaging and Placido disk to provide a complete geometric analysis of the anterior corneal surface, as other commercially available topography systems. To date, there are no scientific studies evaluating the consistency of measurements provided by this new device. The aim of the current study was to evaluate the intrasession repeatability of corneal geometric and aberrometric measurements obtained with this multidiagnostic device in a sample of normal healthy eyes.

## 2. Material and Methods

### 2.1. Patients

In this study of evaluation of a technology, a total of 107 healthy eyes of 107 patients with ages ranging from 23 to 65 years were included. All subjects were randomly selected from patients attending the Optometric Clinic of the University of Alicante, where this investigation was developed. Only one eye from each subject was randomly chosen for the study according to a random number sequence (dichotomic sequence, 0 and 1) in order to avoid the potential interference of the correlation that often exists between the two eyes of the same person. Inclusion criteria were age of more than 18 years, spherical refractive error between +6.00 and −10.00 D, and eyes without pathology. Exclusion criteria were previous ocular surgery, glaucoma, less than 18 complete consecutive Placido rings projected on the cornea and therefore considered for the corneal analysis, pseudophakia, corneal ectatic diseases, and any other type of pathological condition of the eye. All patients were previously informed about the study and signed an informed consent document in accordance with the tenets of the Helsinki Declaration. An approval for the performance of the study was obtained from the Ethics Committee of the University of Alicante (Spain).

### 2.2. Ocular Examination

**Figure 1:**
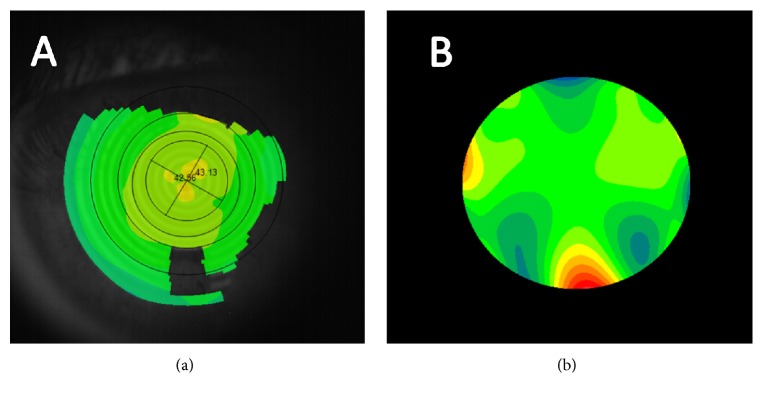
Example of corneal analysis provided by the VX120 system. (a) Axial corneal topographic map. (b) High-order wavefront aberration map.

In all cases, a comprehensive visual and ocular examination was performed, including measurement of uncorrected and best-corrected visual acuity, manifest refraction, air tonometry (VX120 system), and corneal topographic and anterior segment analysis with the multidiagnostic device VX120 (VX120 system). All measurements were performed by a single experienced examiner (ALN) in the period from 10:00 to 13:00 a.m. In all cases, three consecutive measurements were taken with the multidiagnostic system in order to evaluate the intrasession repeatability of corneal curvature, eccentricity, and aberrometric measurements (Figure 1). Specifically, the consistency of the following curvature and geometric parameters were evaluated: keratometric flattest (*K*_flat_) and steepest (*K*_steep_) corneal radius, mean keratometric corneal radius (*K*_*M*_), and mean keratometric astigmatism (*K*_AST_) and its axis (*K*_ASTAX_), corneal eccentricity (*e*), flattest (3*K*_flat_) and steepest (3*K*_steep_) corneal radius, mean corneal radius (3*K*_*M*_), and mean astigmatism (3*K*_AST_) and its axis (3*K*_ASTAX_) in a 3 mm central zone, flattest (5*K*_flat_) and steepest (5*K*_steep_) corneal radius, mean corneal radius (5*K*_*M*_), and mean astigmatism (5*K*_AST_) and its axis (5*K*_ASTAX_) in a 5 mm central zone, and flattest (7*K*_flat_) and steepest (7*K*_steep_) corneal radius, mean corneal radius (7*K*_*M*_), and mean astigmatism (7*K*_AST_) and its axis (7*K*_ASTAX_) in a 7 mm central zone.

Concerning corneal aberrometry, the device provides measurements until the 7th Zernike order. In our study, the following parameters were calculated and evaluated considering a 5 mm pupil aperture: high-order aberration root mean square (RMS) wavefront error, primary coma RMS (*Z*_3_^±1^), primary trefoil RMS (*Z*_3_^±3^), primary spherical aberration Zernike term (*Z*_4_^0^), secondary astigmatism RMS (*Z*_4_^±2^), quadrafoil RMS (*Z*_4_^±4^), secondary coma RMS (*Z*_5_^±1^), secondary trefoil RMS (*Z*_5_^±3^), pentafoil RMS (*Z*_5_^±5^), secondary spherical aberration Zernike term (*Z*_6_^0^), tertiary astigmatism RMS (*Z*_6_^±2^), secondary quadrafoil RMS (*Z*_6_^±4^), hexafoil RMS (*Z*_6_^±6^), tertiary coma RMS (*Z*_7_^±1^), tertiary trefoil RMS (*Z*_7_^±3^), secondary pentafoil RMS (*Z*_7_^±5^), and heptafoil RMS (*Z*_7_^±7^).

### 2.3. The VX120 System

The VX120 system is a multidiagnostic platform that combines a Hartmann-Shack aberrometer, a Placido disk corneal topographer, a Scheimpflug imaging-based system, and an air tonometer. The Placido disk system projects 24 rings on the corneal surface, measuring more than 100,000 points in an area from 0.33 to 10.0 mm, and this information is used to provide all corneal topographic information. The Scheimpflug imaging-based system uses monochromatic blue light of 455 nm to obtain pachymetric measurements with a resolution of ±1 *µ*m, and iridocorneal angle measurements with a resolution of ±1°. This system is able to provide pachymetric measurements in a range from 150 to 1300 *µ*m and iridocorneal angle measurements in a range from 0 to 60°. The Hartmann-Shack aberrometer of the VX120 system measures 1,500 points in 0.2 seconds in an area ranging from 2.0 to 7.0 mm of diameter. It provides in 0.2 seconds a measurement of spherocylindrical refraction (sphere, −20 to 20 D; cylinder, 0 to −8 D; axis, 0 to 180°) and high-order wavefront aberrations. The air tonometer is able to provide intraocular pressure measurements in a range from 1 to 50 mmHg. The combination in one device of all these technologies allows obtaining tangential and axial curvature data of the anterior corneal surface, a biometric estimation of various anterior segment structures, measurement of corneal, internal and ocular wavefront aberrations, visual quality simulations, corneal pachymetry maps, and IOP measurements.

### 2.4. Statistical Analysis

The statistical analysis was performed using the software SPSS version 15.0 for Windows (SPSS, Chicago, Illinois, USA). Normality of all data distributions was confirmed by means of the Kolmogorov-Smirnov test. Then, parametric statistics was applied. Intrasession repeatability for each parameter evaluated was assessed with the following variables: the within-subject standard deviation (*S*_*w*_) of the 3 consecutive measurements, intrasubject precision (1.96 × *S*_*w*_), test-retest repeatability (2.77 × *S*_*w*_), and the intraclass correlation coefficient (ICC). The within-subject standard deviation (*S*_*w*_) is a simple way of estimating the size of the measurement error. The intraobserver precision was defined as (±1.96 × *S*_*w*_) and this parameter indicates how large is the range of error of the repeated measurements for 95% of observations. Finally, the ICC is an ANOVA-based type of correlation that measures the relative homogeneity within groups (between the repeated measurements) in ratio to the total variation. The ICC will approach 1.0 when there is no variance within repeated measurements, indicating total variation in measurements is due solely to variability in the parameter being measured. Furthermore, Pearson correlation coefficients were used to assess the correlation between different parameters evaluated. All statistical tests were 2-tailed, and *p* values less than 0.05 were considered statistically significant.

## 3. Results

A sample of 49 males (45.8%) and 58 females (54.2%), with a mean age of 47.8 years (ranging from 23 to 65 years), was evaluated. Mean refractive sphere in the sample was −0.43 D (standard deviation, SD: 2.02), ranging from −7.00 to +6.00 D. Mean refractive cylinder was −0.40 D (standard deviation, SD: 0.75), ranging from −4.00 to 0.00 D.

Table 1 summarizes the outcomes of the intrasession repeatability analysis for all curvature and eccentricity measurements. As shown, all *S*_*w*_ for corneal power measurements were equal to or below 0.25 D, with ICC ranging from 0.982 to 0.995. The *S*_*w*_ for the magnitude of corneal astigmatism calculated for different areas of analysis was below 0.21 D, with ICC ranging from 0.913 to 0.976. Concerning the axis of astigmatism, its *S*_*w*_ was below 5.76 degrees, with ICC ranging from 0.975 to 0.995.  Table 2 shows the differences in *S*_*w*_ for the magnitude of corneal astigmatism and axis according to the magnitude of corneal astigmatism (≤0.75 and >0.75 D). As shown, significantly larger variability of *S*_*w*_ was found in the axis of corneal astigmatism measured at different corneal areas for astigmatisms of 0.75 D or lower (*p* ≤ 0.002). Likewise, the difference in *S*_*w*_ for the magnitude of corneal astigmatism between eyes with corneal astigmatism ≤0.75 D and those with astigmatisms of more than 0.75 D was small in magnitude but statistically significant (*p* ≤ 0.002), with the larger values for those eyes with more astigmatism. Corneal eccentricity showed *S*_*w*_ and ICC of 0.067 and 0.957, respectively.

Table 3 summarizes the outcomes of the intrasession repeatability analysis for corneal aberrometric measurements (5 mm pupil). As shown, *S*_*w*_ and ICC of 0.048 *µ*m and 0.901 were obtained, respectively, for HOA RMS. For the aberrometric parameters of the third- and fourth-order, all *S*_*w*_ were below 0.037 *µ*m and all ICC were higher than 0.84, except for quadrafoil RMS that had an ICC associated of 0.689. For the fifth-, sixth-, and seventh-order parameters, *S*_*w*_ ranged from 0.005 *µ*m for tertiary coma RMS to 0.018 *µ*m for pentafoil RMS, and ICC ranged from 0.424 for tertiary trefoil RMS to 0.845 for tertiary astigmatism RMS.

**Figure 2:**
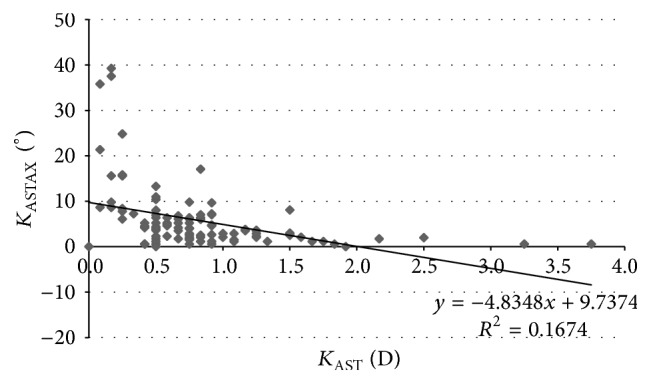
Scatterplot showing the relationship among the mean magnitude of keratometric corneal astigmatism (*K*_AST_) and the within-subject standard deviation (*S*_*w*_) associated with the axis of such corneal astigmatism (*K*_ASTAX_). The adjusting line of data obtained by means of the least-squares fit is displayed.

**Figure 3:**
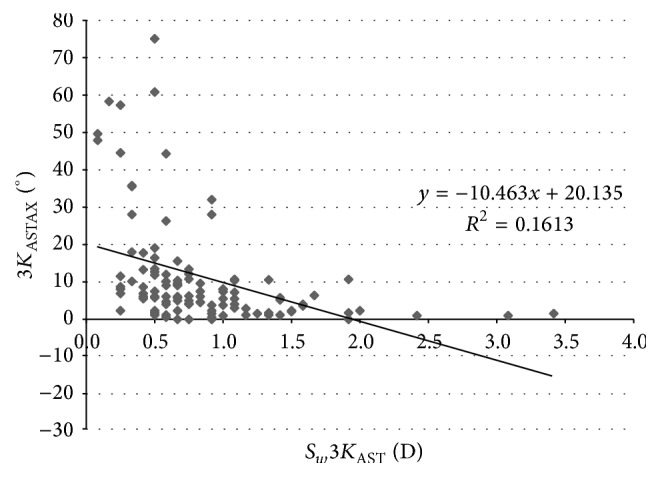
Scatterplot showing the relationship among the mean magnitude of corneal astigmatism in the 3 mm central zone (3*K*_AST_) and the within-subject standard deviation (*S*_*w*_) associated with the axis of such corneal astigmatism (3*K*_ASTAX_). The adjusting line of data obtained by means of the least-squares fit is displayed.

**Figure 4:**
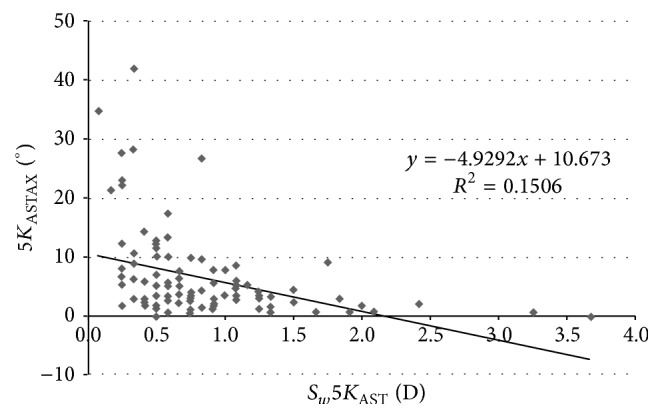
Scatterplot showing the relationship among the mean magnitude of corneal astigmatism in the 5 mm central zone (5*K*_AST_) and the within-subject standard deviation (*S*_*w*_) associated with the axis of such corneal astigmatism (5*K*_ASTAX_). The adjusting line of data obtained by means of the least-squares fit is displayed.

**Figure 5:**
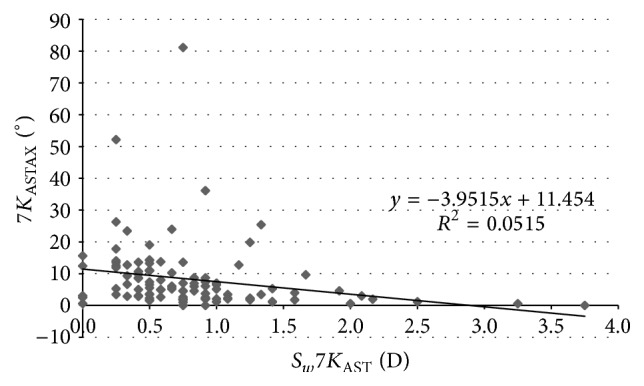
Scatterplot showing the relationship among the mean magnitude of corneal astigmatism in the 7 mm central zone (7*K*_AST_) and the within-subject standard deviation (*S*_*w*_) associated with the axis of such corneal astigmatism (7*K*_ASTAX_). The adjusting line of data obtained by means of the least-squares fit is displayed.

Table 4 displays the coefficients of correlation of all relationships between different corneal power and eccentricity parameters evaluated and their *S*_*w*_ associated. As shown, statistically significant correlations were found between some corneal power parameters and their *S*_*w*_ values associated, but these correlations were very weak. Moderate and statistically significant correlations were found between the magnitude of astigmatism and the *S*_*w*_ associated with the axis of astigmatism for the different corneal areas analyzed (*K*_AST_ − *S*_*w*_*K*_ASTAX_, *r* = −0.409, *p* < 0.001 (Figure 2); 3*K*_AST_ − *S*_*w*_3*K*_ASTAX_, *r* = −0.402, *p* < 0.001 (Figure 3); 5*K*_AST_ − *S*_*w*_5*K*_ASTAX_, *r* = −0.388, *p* < 0.001 (Figure 4); 7*K*_AST_ − *S*_*w*_7*K*_ASTAX_, *r* = −0.242, *p* = 0.012 (Figure 5)).

Concerning the corneal aberrometric data, statistically significant positive correlations were found between the magnitude of the aberrometric parameters evaluated and their *S*_*w*_ (*r* ≥ 0.375, *p* < 0.001), except for the Zernike term corresponding to the secondary spherical aberration (*r* = −0.014, *p* = 0.886) (Table 5). The strongest correlations were found for secondary trefoil (*r* = 0.708), pentafoil (*r* = 0.714), and secondary pentafoil RMS (*r* = 0.715) (Table 4).

## 4. Discussion

New multidiagnostic devices combining Scheimpflug imaging and other technologies provide a complete analysis of corneal structure, including a large variety of anatomical and optical parameters [1]. There are scientific evidence confirming the consistency of corneal measurements provided by different commercially available multidiagnostic devices based on Scheimpflug imaging [2–6]. The VX120 is a new multidiagnostic platform combining Scheimpflug imaging with Placido disk, Hartmann-Shack, and air-puff tonometry technologies. To date, no studies have been reported evaluating the level of consistency of corneal anatomical and optical properties measured with this device. The purpose of the current study was to evaluate the intrasession repeatability of corneal geometric and aberrometric measurements provided by this new device in a normal healthy population.

In our study, the intrasession repeatability of the keratometric measurements as well as of corneal power measurements at 3, 5, and 7 mm was excellent, with *S*_*w*_ values below 0.26 D and ICC of more than 0.98. These intrasession repeatability outcomes are similar to those reported with other commercially available Scheimpflug imaging-based systems [2–14]. Montalbán et al. [6] found *S*_*w*_ values of 0.4 mm or lower (~0.20 D) and ICC of more than 0.990 for repeated measurements of anterior and posterior corneal curvature obtained in normal healthy eyes with the Sirius system from CSO (Firenze, Italy). These same authors also reported slightly poorer intrasession consistency of corneal curvature measurements with the same system in keratoconus eyes [5]. Cerviño et al. [2] reported ICC values of more than 0.950 for anterior and posterior corneal curvature measurements obtained in normal healthy eyes with the Galilei dual Scheimpflug-Placido analyzer from Ziemer Ophthalmic Systems AG (Switzerland). Kim et al. [7] reported for the same dual Scheimpflug-Placido system *S*_*w*_ values of 0.08 and 0.09 D for simulated keratometry in normal healthy and postrefractive surgery eyes, respectively. McAlinden et al. [12] reported repeatability limits expressed as the within-subject standard deviation × $1.96\sqrt{2}$ of the anterior flattest and steepest keratometry readings of 0.25 and 0.36 D, respectively, using the Pentacam system from Oculus (Wetzlar, Germany) in a sample of normal healthy eyes.

In our study, the *S*_*w*_ values for corneal astigmatism at central 3, 5, and 7 mm were below 0.21 D and ICC was of more than 0.913. This level of consistency is similar or even better than that reported for other commercially available multidiagnostic platforms [2, 5, 6, 15, 16]. Cerviño et al. [2] found an ICC value of 0.811 for the magnitude of corneal astigmatism obtained in normal healthy eyes with the Galilei dual Scheimpflug-Placido analyzer. Similarly, moderate ICC values were found by Masoud et al. [15] for corneal astigmatism in normal healthy eyes using the Sirius system. In some studies, corneal astigmatism was converted into astigmatic power vector components considering the magnitude and axis, *J*_0_ and *J*_45_, and the repeatability of such vectors has been analyzed. Montalbán et al. [5, 6] found excellent repeatability for the power vector components of anterior corneal astigmatism measured with the Sirius system in normal healthy and keratoconus eyes. Regarding the axis of corneal astigmatism, *S*_*w*_ was below 5.8 degrees and ICC was over 0.975 in our sample. Although the VX120 system provided consistent data of astigmatism axis in most of cases, significant variability was observed for the axis of corneal astigmatisms of small magnitude. Indeed, significantly higher values of *S*_*w*_ for the axis of corneal astigmatism were found in those eyes with corneal astigmatisms ≤0.75 D. Likewise, weak to moderate statistically significant correlations were found between the magnitude of corneal astigmatism and the *S*_*w*_ associated with the axis of astigmatism. This relative limitation of corneal topographers of obtaining consistent measurements of the axis of very low corneal astigmatisms has been studied before by Fityo et al. [17].

The consistency of corneal eccentricity measurements obtained with the VX120 system in our study was also good, with *S*_*w*_ and ICC of 0.067 and 0.957, respectively. This is consistent with the results of other studies evaluating the intrasession repeatability of shape factor or corneal eccentricity measurements obtained with other Scheimpflug imaging-based systems [5, 6, 8, 18]. Savini et al. [18] obtained ICC values of 0.904 and 0.977 for anterior and posterior corneal asphericity measurements using the Sirius system in normal healthy eyes.

Concerning corneal aberrometric measurements (5 mm pupil), high levels of intrasession repeatability were found for HOA RMS (*S*_*w*_: 0.048 *µ*m, ICC: 0.901) as well as for third- and fourth-order aberrometric parameters (*S*_*w*_ < 0.037 *µ*m and ICC > 0.84), except for quadrafoil RMS (ICC: 0.689). Bayhan et al. [19] reported somewhat lower level of consistency of anterior corneal aberrometric measurements using the Sirius system in normal healthy eyes (ICC from 0.568 for quadrafoil RMS to 0.856 for primary coma RMS). Similarly, Cerviño et al. [2] reported a moderate to good consistency of corneal aberrometric measurements obtained in normal healthy eyes using the Galilei system. Wang et al. [13] reported good consistency of measurements of third- and fourth-order aberrometric parameters obtained with the Galilei system in healthy eyes, with *S*_*w*_ values of 0.08, 0.09, 0.02, 0.04, and 0.09 *µ*m for primary coma, trefoil, primary spherical aberration, secondary astigmatism, and quadrafoil, respectively. In our study, the consistency was more limited for fifth-, sixth-, and seventh-order aberrometric parameters, with *S*_*w*_ ranging from 0.005 *µ*m for tertiary coma RMS to 0.018 *µ*m for pentafoil RMS and ICC ranging from 0.424 for tertiary trefoil RMS to 0.845 for tertiary astigmatism RMS.

Finally, moderate and statistically significant positive correlations were found among the magnitude of aberrometric parameters and the *S*_*w*_ values associated with them, except for the Zernike term corresponding to the secondary spherical aberration. This suggests that the consistency of aberrometric measurements may be limited in eyes with significant amounts of high-order aberrations. However, in spite of these correlations, the ranges of variability of the aberrometric measurements in our study were not clinically relevant, even in those eyes with larger amounts of higher order aberrations. Future studies should be conducted to evaluate the consistency of aberrometric measurements obtained with the VX120 system in highly aberrated corneas, such as keratoconus. In a previous study, a limitation by the level of aberration of some internal aberrometric parameters (subtraction of corneal to total aberrations) measured with an integrated system combining a Hartmann-Shack aberrometer and a Placido disk corneal topographer has been reported [20].

One limitation of this study is that interobserver repeatability and interchangeability analyses have not been done. The results of this study have only demonstrated that the multidiagnostic platform evaluated is able to provide repeatable measurements of different geometric and aberrometric corneal parameters, which is crucial for an instrument to be used in clinical practice. However, future studies are necessary to evaluate the interobserver repeatability and the interchangeability of the measurements provided by the VX120 platform and other commercially available systems. In any case, no much difference is expected to be found with the analysis of the interobserver repeatability compared to intrasession repeatability as measurements are taken automatically by the VX120 system, with minimal intervention from the observer. Finally, the evaluation of the repeatability of corneal measurements obtained with the VX120 system in pathological eyes, such as keratoconus, should be also done in future studies.

In conclusion, the multidiagnostic system VX120 is able to provide consistent measurements of corneal power at different areas, eccentricity, and third- and fourth-order aberrations in healthy eyes. The consistency of corneal power and eccentricity measurements is not dependent on the magnitude of the measurement, with the same precision ability for flat and steep corneas within the normal range. More variability is present for fifth- to seventh-order aberrations as well as for the measurement of the axis of low corneal cylinders. For these parameters, it is recommendable to consider the average of several consecutive measurements.

## Figures and Tables

**Table 1:** Summary of the intrasession repeatability outcomes for the corneal curvature and eccentricity measurements obtained by means of the VX120 system.

	Overall mean (SD) Overall median (range)	*S*_*w*_	1.96 × *S*_*w*_	2.77 × *S*_*w*_	ICC (range 95% CI)
*K*_flat_ (mm)	42.28 (1.61)	0.19	0.37	0.52	0.993
	42.38 (37.63 to 45.98)				(0.990 to 0.995)
*K*_steep_ (mm)	43.11 (1.64)	0.20	0.39	0.55	0.993
	43.31 (38.63 to 47.01)				(0.990 to 0.995)
*K*_*M*_ (mm)	42.69 (1.60)	0.18	0.35	0.50	0.993
	42.96 (38.19 to 46.49)				(0.991 to 0.995)
*K*_AST_ (D)	0.82 (0.59)	0.11	0.21	0.30	0.976
	0.75 (0.00 to 3.75)				(0.967 to 0.983)
*K*_ASTAX_ (degrees)	102.96 (72.17)	5.76	11.29	15.95	0.995
	136.67 (1 a 180)				(0.993 to 0.996)
*e*	0.34 (0.31)	0.07	0.13	0.19	0.957
	0.43 (−1.16 to 0.83)				(0.941 to 0.969)
3*K*_flat_ (mm)	42.03 (1.63)	0.25	0.49	0.69	0.988
	42.21 (37.18 to 45.75)				(0.984 to 0.992)
3*K*_steep_ (mm)	42.89 (1.66)	0.25	0.49	0.69	0.989
	43.09 (37.99 to 46.96)				(0.985 to 0.992)
3*K*_*M*_ (mm)	42.46 (1.62)	0.22	0.44	0.62	0.991
	42.65 (37.89 to 46.34)				(0.987 to 0.993)
3*K*_AST_ (D)	0.85 (0.56)	0.20	0.40	0.56	0.913
	0.67 (0.08 to 3.42)				(0.879 to 0.938)
3*K*_ASTAX_ (degrees)	85.81 (67.43)	5.76	11.28	15.95	0.975
	85.00 (0 to 180)				(0.966 to 0.982)
5*K*_flat_ (mm)	42.11 (1.60)	0.20	0.39	0.55	0.992
	42.28 (37.57 to 45.79)				(0.989 to 0.994)
5*K*_steep_ (mm)	42.94 (1.62)	0.21	0.41	0.58	0.992
	43.12 (38.69 to 46.90)				(0.989 to 0.994)
5*K*_*M*_ (mm)	42.52 (1.59)	0.19	0.37	0.52	0.993
	42.79 (38.22 to 46.34)				(0.990 to 0.995)
5*K*_AST_ (D)	0.82 (0.58)	0.145	0.283	0.40	0.960
	0.67 (0.08 to 3.67)				(0.944 to 0.971)
5*K*_ASTAX_ (degrees)	91.83 (70.67)	6.653	13.041	18.43	0.993
	97.67 (0 to 180)				(0.991 to 0.995)
7*K*_flat_ (mm)	42.03 (1.55)	0.20	0.40	0.56	0.987
	42.12 (37.68 to 45.67)				(0.982 to 0.991)
7*K*_steep_ (mm)	42.86 (1.57)	0.19	0.36	0.52	0.993
	43.01 (39.26 to 46.79)				(0.991 to 0.995)
7*K*_*M*_ (mm)	42.44 (1.53)	0.18	0.35	0.50	0.992
	42.56 (38.70 to 46.21)				(0.989 to 0.994)
7*K*_AST_ (D)	0.83 (0.59)	0.139	0.273	0.39	0.923
	0.75 (0.25 to 3.75)				(0.893 to 0.945)
7*K*_ASTAX_ (degrees)	88.82 (70.73)	8.247	16.164	22.84	0.988
	91.67 (0 to 180)				(0.984 to 0.992)

SD, standard deviation; keratometric flattest (*K*_flat_) and steepest (*K*_steep_) corneal radius, mean keratometric corneal radius (*K*_*M*_), and mean keratometric astigmatism (*K*_AST_) and its axis (*K*_ASTAX_), corneal eccentricity (*e*), flattest (3*K*_flat_) and steepest (3*K*_steep_) corneal radius, mean corneal radius (3*K*_*M*_), and mean astigmatism (3*K*_AST_) and its axis (3*K*_ASTAX_) in a 3 mm central zone, flattest (5*K*_flat_) and steepest (5*K*_steep_) corneal radius, mean corneal radius (5*K*_*M*_), and mean astigmatism (5*K*_AST_) and its axis (5*K*_ASTAX_) in a 5 mm central zone, and flattest (7*K*_flat_) and steepest (7*K*_steep_) corneal radius, mean corneal radius (7*K*_*M*_), and mean astigmatism (7*K*_AST_) and its axis (7*K*_ASTAX_) in a 7 mm central zone.

**Table 2:** Summary of the intrasession repeatability outcomes for the corneal astigmatism and axis measurements obtained by means of the VX120 system depending on the magnitude of corneal astigmatism.

*S*_*w*_	Corneal astigmatism ≤0.75 D	Corneal astigmatism >0.75 D	*p* value
*K*_AST_ (D)	0.10	0.13	0.148
*K*_ASTAX_ (°)	7.25	3.44	0.001
3*K*_AST_ (D)	0.15	0.28	0.001
3*K*_ASTAX_ (°)	14.72	5.92	0.001
5*K*_AST_ (D)	0.11	0.19	0.002
5*K*_ASTAX_ (°)	8.25	4.19	0.002
7*K*_AST_ (D)	0.13	0.15	0.090
7*K*_ASTAX_ (°)	9.43	6.42	0.002

Mean keratometric astigmatism (*K*_AST_) and its axis (*K*_ASTAX_), mean astigmatism (3*K*_AST_) and its axis (3*K*_ASTAX_) in a 3 mm central zone, mean astigmatism (5*K*_AST_) and its axis (5*K*_ASTAX_) in a 5 mm central zone, and mean astigmatism (7*K*_AST_) and its axis (7*K*_ASTAX_) in a 7 mm central zone.

**Table 3:** Summary of the intrasession repeatability outcomes for the corneal aberrometric measurements obtained by means of the VX120 system.

	Overall mean (SD) Overall median (range)	*S*_*w*_	Pr	2.77 × *S*_*w*_	ICC (range 95% CI)
HOA RMS (*µ*m)	0.25 (0.11)	0.048	0.093	0.13	0.901
	0.23 (0.06 to 0.73)				(0.864 to 0.930)
Primary coma RMS (*Z*_3_^±1^)	0.14 (0.08)	0.031	0.062	0.09	0.916
	0.13 (0.03 to 0.49)				(0.885 to 0.941)
Primary trefoil RMS (*Z*_3_^±3^)	0.11 (0.07)	0.036	0.070	0.10	0.845
	0.10 (0.02 to 0.42)				(0.787 to 0.890)
Primary spherical aberration Zernike term (*Z*_4_^0^)	0.10 (0.08)	0.021	0.041	0.06	0.958
	0.08 (0.02 to 0.63)				(0.942 to 0.970)
Secondary astigmatism RMS (*Z*_4_^±2^)	0.04 (0.03)	0.014	0.027	0.04	0.887
	0.03 (0.01 to 0.19)				(0.844 to 0.919)
Quadrafoil RMS (*Z*_4_^±4^)	0.06 (0.03)	0.025	0.048	0.07	0.689
	0.05 (0.01 to 0.21)				(0.571 to 0.689)
Secondary coma RMS (*Z*_5_^±1^)	0.02 (0.01)	0.008	0.015	0.02	0.679
	0.01 (0.00 to 0.06)				(0.557 to 0.772)
Secondary trefoil RMS (*Z*_5_^±3^)	0.02 (0.01)	0.010	0.020	0.03	0.564
	0.02 (0.00 to 0.06)				(0.398 to 0.690)
Pentafoil RMS (*Z*_5_^±5^)	0.04 (0.02)	0.018	0.036	0.05	0.473
	0.04 (0.01 to 0.16)				(0.273 to 0.625)
Secondary spherical aberration Zernike term (*Z*_6_^0^)	−0.01 (0.01)	0.007	0.014	0.02	0.724
	−0.01 (−0.06 to 0.04)				(0.620 to 0.804)
Tertiary astigmatism RMS (*Z*_6_^±2^)	0.01 (0.01)	0.007	0.013	0.02	0.845
	0.01 (0.00 to 0.12)				(0.786 to 0.890)
Secondary quadrafoil RMS (*Z*_6_^±4^)	0.02 (0.01)	0.011	0.021	0.03	0.617
	0.02 (0.01 to 0.10)				(0.471 to 0.727)
Hexafoil RMS (*Z*_6_^±6^)	0.04 (0.02)	0.017	0.032	0.05	0.590
	0.04 (0.01 to 0.12)				(0.434 to 0.708)
Tertiary coma RMS (*Z*_7_^±1^)	0.01 (0.01)	0.005	0.011	0.01	0.516
	0.01 (0.00 to 0.03)				(0.332 to 0.656)
Tertiary trefoil RMS (*Z*_7_^±3^)	0.01 (0.01)	0.006	0.013	0.02	0.424
	0.01 (0.00 to 0.04)				(0.205 to 0.590)
Secondary pentafoil RMS (*Z*_7_^±5^)	0.02 (0.01)	0.009	0.018	0.03	0.623
	0.02 (0.00 to 0.09)				(0.480 to 0.732)
Heptafoil RMS (*Z*_7_^±7^)	0.03 (0.01)	0.011	0.022	0.03	0.633
	0.03 (0.01 to 0.08)				(0.493 to 0.739)

SD, standard deviation; HOA, high order aberrations; RMS, root mean square.

**Table 4:** Summary of correlations between different corneal power and eccentricity parameters and their within-subject standard deviation (*S*_*w*_) associated.

	Correlation Pearson coefficient	*p* value
*K*_flat_ (mm)	0.269	0.005
*K*_steep_ (mm)	0.178	0.066
*K*_*M*_ (mm)	0.276	0.004
*K*_AST_ (D)	−0.077	0.431
*K*_ASTAX_ (degrees)	0.052	0.597
*e*	−0.244	0.011
3*K*_flat_ (mm)	0.125	0.200
3*K*_steep_ (mm)	0.053	0.588
3*K*_*M*_ (mm)	0.140	0.151
3*K*_AST_ (D)	0.269	0.005
3*K*_ASTAX_ (degrees)	−0.051	0.603
5*K*_flat_ (mm)	0.215	0.026
5*K*_steep_ (mm)	0.129	0.184
5*K*_*M*_ (mm)	0.240	0.013
5*K*_AST_ (D)	0.218	0.024
5*K*_ASTAX_ (degrees)	0.133	0.173
7*K*_flat_ (mm)	0.166	0.087
7*K*_steep_ (mm)	0.207	0.033
7*K*_*M*_ (mm)	−0.128	0.189
7*K*_AST_ (D)	0.125	0.201
7*K*_ASTAX_ (degrees)	0.075	0.445

SD, standard deviation; keratometric flattest (*K*_flat_) and steepest (*K*_steep_) corneal radius, mean keratometric corneal radius (*K*_*M*_), and mean keratometric astigmatism (*K*_AST_) and its axis (*K*_ASTAX_), corneal eccentricity (*e*), flattest (3*K*_flat_) and steepest (3*K*_steep_) corneal radius, mean corneal radius (3*K*_*M*_), and mean astigmatism (3*K*_AST_) and its axis (3*K*_ASTAX_) in a 3 mm central zone, flattest (5*K*_flat_) and steepest (5*K*_steep_) corneal radius, mean corneal radius (5*K*_*M*_), and mean astigmatism (5*K*_AST_) and its axis (5*K*_ASTAX_) in a 5 mm central zone, and flattest (7*K*_flat_) and steepest (7*K*_steep_) corneal radius, mean corneal radius (7*K*_*M*_), and mean astigmatism (7*K*_AST_) and its axis (7*K*_ASTAX_) in a 7 mm central zone.

**Table 5:** Summary of correlations between different corneal aberrometric parameters and their within-subject standard deviation (*S*_*w*_) associated.

	Correlation Pearson coefficient	*p* value
HOA RMS (*µ*m)	0.451	<0.001
Primary coma RMS (*Z*_3_^±1^)	0.518	<0.001
Primary trefoil RMS (*Z*_3_^±3^)	0.529	<0.001
Primary spherical aberration Zernike term (*Z*_4_^0^)	0.507	<0.001
Secondary astigmatism RMS (*Z*_4_^±2^)	0.523	<0.001
Quadrafoil RMS (*Z*_4_^±4^)	0.394	<0.001
Secondary coma RMS (*Z*_5_^±1^)	0.562	<0.001
Secondary trefoil RMS (*Z*_5_^±3^)	0.708	<0.001
Pentafoil RMS (*Z*_5_^±5^)	0.714	<0.001
Secondary spherical aberration Zernike term (*Z*_6_^0^)	−0.014	0.886
Tertiary astigmatism RMS (*Z*_6_^±2^)	0.511	<0.001
Secondary quadrafoil RMS (*Z*_6_^±4^)	0.487	<0.001
Hexafoil RMS (*Z*_6_^±6^)	0.514	<0.001
Tertiary coma RMS (*Z*_7_^±1^)	0.375	<0.001
Tertiary trefoil RMS (*Z*_7_^±3^)	0.487	<0.001
Secondary pentafoil RMS (*Z*_7_^±5^)	0.715	<0.001
Heptafoil RMS (*Z*_7_^±7^)	0.554	<0.001

SD, standard deviation; HOA, high order aberrations; RMS, root mean square.
